# Impact of collimator leaf width and treatment technique on stereotactic radiosurgery and radiotherapy plans for intra- and extracranial lesions

**DOI:** 10.1186/1748-717X-4-3

**Published:** 2009-01-21

**Authors:** Q Jackie Wu, Zhiheng Wang, John P Kirkpatrick, Zheng Chang, Jeffrey J Meyer, Mei Lu, Calvin Huntzinger, Fang-Fang Yin

**Affiliations:** 1 Department of Radiation Oncology, Duke University, Durham, NC, USA; 2 Department of Biostatistics and Research Epidemiology, Henry Ford Health System, Detroit, MI, USA; 3 Varian Medical Systems, Palo Alto, CA, USA

## Abstract

**Background:**

This study evaluated the dosimetric impact of various treatment techniques as well as collimator leaf width (2.5 vs 5 mm) for three groups of tumors – spine tumors, brain tumors abutting the brainstem, and liver tumors. These lesions often present challenges in maximizing dose to target volumes without exceeding critical organ tolerance. Specifically, this study evaluated the dosimetric benefits of various techniques and collimator leaf sizes as a function of lesion size and shape.

**Methods:**

Fifteen cases (5 for each site) were studied retrospectively. All lesions either abutted or were an integral part of critical structures (brainstem, liver or spinal cord). For brain and liver lesions, treatment plans using a 3D-conformal static technique (3D), dynamic conformal arcs (DARC) or intensity modulation (IMRT) were designed with a conventional linear accelerator with standard 5 mm leaf width multi-leaf collimator, and a linear accelerator dedicated for radiosurgery and hypofractionated therapy with a 2.5 mm leaf width collimator. For the concave spine lesions, intensity modulation was required to provide adequate conformality; hence, only IMRT plans were evaluated using either the standard or small leaf-width collimators.

A total of 70 treatment plans were generated and each plan was individually optimized according to the technique employed. The Generalized Estimating Equation (GEE) was used to separate the impact of treatment technique from the MLC system on plan outcome, and t-tests were performed to evaluate statistical differences in target coverage and organ sparing between plans.

**Results:**

The lesions ranged in size from 2.6 to 12.5 cc, 17.5 to 153 cc, and 20.9 to 87.7 cc for the brain, liver, and spine groups, respectively. As a group, brain lesions were smaller than spine and liver lesions. While brain and liver lesions were primarily ellipsoidal, spine lesions were more complex in shape, as they were all concave. Therefore, the brain and the liver groups were compared for volume effect, and the liver and spine groups were compared for shape. For the brain and liver groups, both the radiosurgery MLC and the IMRT technique contributed to the dose sparing of organs-at-risk(OARs), as dose in the high-dose regions of these OARs was reduced up to 15%, compared to the non-IMRT techniques employing a 5 mm leaf-width collimator. Also, the dose reduction contributed by the fine leaf-width MLC decreased, as dose savings at all levels diminished from 4 – 11% for the brain group to 1 – 5% for the liver group, as the target structures decreased in volume. The fine leaf-width collimator significantly improved spinal cord sparing, with dose reductions of 14 – 19% in high to middle dose regions, compared to the 5 mm leaf width collimator.

**Conclusion:**

The fine leaf-width MLC in combination with the IMRT technique can yield dosimetric benefits in radiosurgery and hypofractionated radiotherapy. Treatment of small lesions in cases involving complex target/OAR geometry will especially benefit from use of a fine leaf-width MLC and the use of IMRT.

## Background

Stereotactic intracranial radiosurgery (SRS) and extracranial body radiosurgery and radiotherapy (SBRT) are characterized by ablative, high dose irradiation of target structures. Complex targets, such as spine metastases, brain lesions abutting the brain stem, and liver lesions present challenges in maximizing the dose to the target volume while not exceeding the critical organ tolerances [1-20]. This study evaluates the benefits of a dedicated radiosurgery system with a fine leaf-width collimator for these different groups of patients. More specifically, this study evaluates the dosimetric benefits of this system as a function of target size and shape complexity.

## Materials and methods

### Patient Data

**Figure 1:**
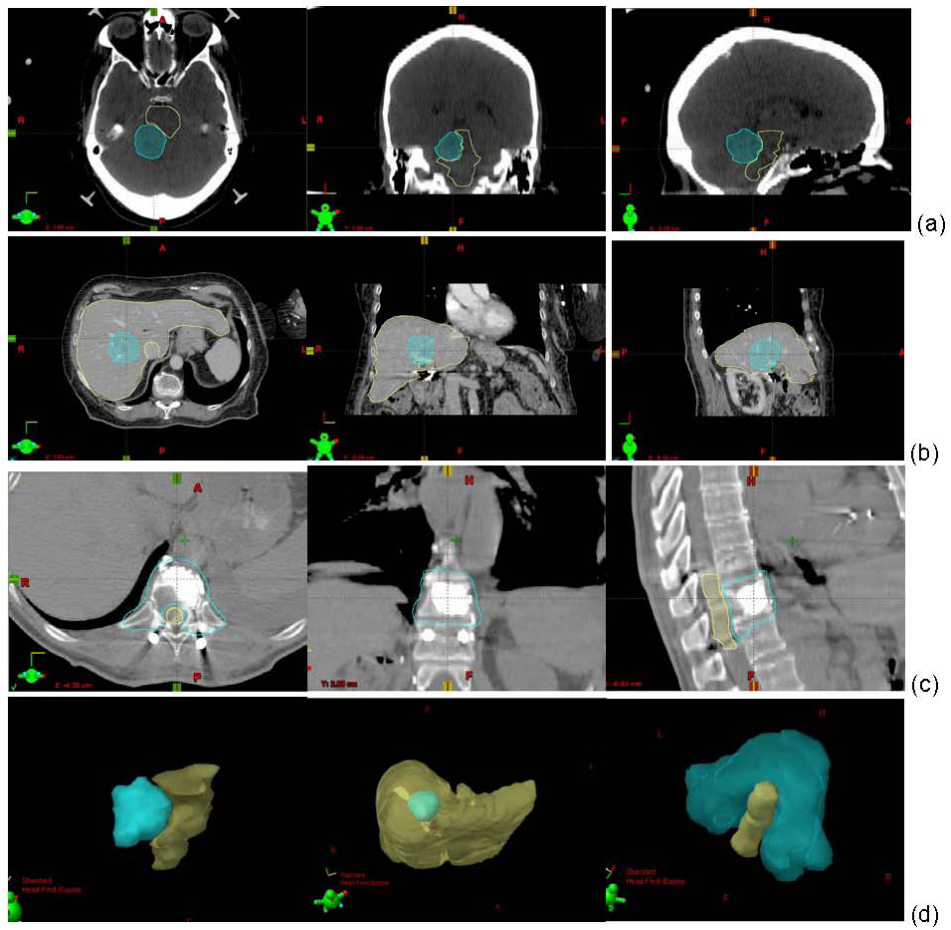
**Examples of lesions and adjacent critical structures/organs for the brain (a), the liver(b), and the spine (c) groups**. The 3D rendering of the geometrical relationships is shown in (d) for the brain case (left), liver case (middle) and spine case (right).

This retrospective study included 15 cases (5 each for brain, liver and spine sites) treated with SRS/SBRT at our institution. As shown in Additional file 1, these lesions ranged in size from 2.6 – 12.5 cc, 17.5 – 153.1 cc, and 20.9 – 87.7 cc for the brain, liver, and spine groups, respectively. The volumes selected were intended to represent the ranges of the target volumes typically encountered for these sites. The selected cases all involved lesions next to or within critical structures, *i.e*., lesions next to the brainstem for the brain group, lesions within the liver for the liver group, and tumors near/abutting the spinal cord for the spine group. The brain and liver lesions were mostly ellipsoidal, with brain lesions having smaller volumes. The spine lesions were more complex in shape, as they were all concave; volumes were similar to the liver lesions. Therefore, the brain and the liver groups were compared for volume effect and the liver and the spine groups were compared for shape effect. Figure 1 displays the rendering of the target volumes and critical structures in 3D space for these three sites.

### Treatment planning

Treatment plans were designed with the Novalis Tx radiosurgery system (BrainLab AG, Munich, Germany and Varian Medical Systems, Palo Alto, CA) versus the standard Varian 2100Clinac system (Varian Medical Systems, Palo Alto, CA), which served as the baseline for comparison. The Novalis Tx radiosurgery system became commercially available in early 2008, and was equipped with a newly designed micro-mulitleaf collimator (HD120 MLC) system, replacing the standard Millennium MLC system. In contrast to previous micro-MLC systems [2,21-24], the Novalis Tx radiosurgery MLC is within the gantry housing, instead of being an add-on tertiary system [2,21-24]. The mounting of an add-on tertiary MLC not only prolongs the treatment procedure, but also reduces the clearance between the gantry and couch, therefore limiting the freedom to select certain beam angles. The Novalis Tx HD120 MLC has 32 leaf pairs in the center, each a with leaf width of 2.5 mm (projected at the isocenter) and 14 leaf pairs on each side (a total of 28) with a leaf width of 5 mm; thus, the total number of leaves is 120. Our initial measurements also showed a sharper penumbra. The HD120 MLC leaf side penumbra was 2.5 mm vs. 2.8 mm with the standard MLC, and the HD120 MLC leaf end penumbra was 2.8 mm vs. 3.6 mm with the standard MLC[25]. For conformal static treatment (3D) and dynamic conformal arc treatment (DARC) treatments, the maximum dose rate with the Novalis Tx system is 1000 MU/min versus 600 MU/min with the Clinac system, allowing faster radiation delivery.

For each case, the normal structures and OARs were contoured by a physician with expertise in SRS/SBRT. The target volumes were contoured by the same physician. For the brain lesions, the planning target volume (PTV) was generated by expanding the contrast-enhancing T1-weighted MRI volume by 1-mm, except at the junctions of the tumor and brainstem where no expansion was added. The liver lesions were treated with deep inhale breath-hold technique and cone-beam CT (CBCT) guidance, and the PTV was obtained by expanding the lesion volume by 5 mm right-left and anterior-posterior and 7 mm superior-inferior. The PTV expansion for spine lesions was also non-uniform, usually 3 mm except at the lesion and cord interface, where 0 – 1 mm expansion was used.

Treatment plans were generated using three techniques: 3D, DARC, and IMRT. For the brain and liver lesions, plans using all three techniques were designed. For each lesion and planning technique, plans were generated using both the standard MLC and the HD120 MLC. All the spine lesions exhibited concave shapes, and the 3D and the DARC techniques could not provide clinically adequate conformality. Hence, only IMRT plans were generated for the spine lesions, again using both the standard MLC and HD120 MLC. Typically, the 3D plans used 6–12 beams, DARC plans used 4 – 7 arcs and IMRT plans used 4–12 static beams. The multiple arcs for the brain lesions were mainly designed to take advantage of different couch angles and those for the liver lesions utilized different beam weightings through different sections of anatomy. Each plan was individually optimized according to the treatment techniques selected. Beam angles (non-coplanar or coplanar) were chosen to minimize doses to the critical structures and to achieve high dose fall-off around the target at the same time. For IMRT planning, planning objectives included dose uniformity to the PTV and dose constraints for the OARs as well as dose falloff at the target boundary. For each plan, 90% of the prescription dose covered at least 97% of the PTV.

In summary, a total of 70 treatment plans were designed and each plan was individually optimized according to the technique employed. For the purpose of the dosimetric analysis in this study, the prescription dose was set to a nominal 12.5 Gy in a single fraction for the brain lesions, three 12-Gy fractions totaling 36 Gy for the liver lesions, and 18 Gy in a single fraction for the spine lesions, respectively.

### Dosimetric evaluation parameters and statistical analysis

Each treatment plan was evaluated with respect to target coverage criteria and OAR sparing criteria. For targets, the mean PTV doses, as well as the minimum and maximum doses to the PTVs, were compared. The maximum dose (D_max_) was defined as the maximum dose value that covers 1% of the target volume (i.e. D_1_) and the minimum dose (D_min_) was defined as the minimum dose value that covers 99% of the target volume (i.e. D_99_). The dosimetric metrics for the OARs were D_max_, D_mean_, D_5 _and D_1 _for the brainstem; D_mean_, D_10 _and D_30 _for the liver; and D_max_, D_mean _and D_10 _for the spine cases, respectively.

We generalized the shape into two basic categories: the round/ellipsoidal shapes which are representative of all the targets in the brain and the liver group, and the concave shapes which are representative of all the spine cases, as shown in figure 1. We also generalized the volumes into two major categories, the small volume sizes which are generally seen in the intracranial group and the large volume sizes which are generally seen in the extracranial group, as shown in Additional file 1.

Both the treatment technique and the MLC system can impact the dosimetric outcome for the brain and the liver groups. Therefore, the Generalized Estimating Equation technique (GEE) [26,27] was used to separate their individual influences and to analyze their interactions for dosimetry outcomes. The analysis started by testing for the two-factor interaction, followed either by testing for the individual factor effect if no interaction was detected at critical value of 0.05, or by conducting the pair-wise group comparison if the interaction was significant at the 0.05 level. For the spine lesions, a paired-t test was performed to evaluate the difference between the HD120 MLC and the standard MLC on dosimetric parameters for IMRT.

## Results

### PTV coverage

**Figure 2:**
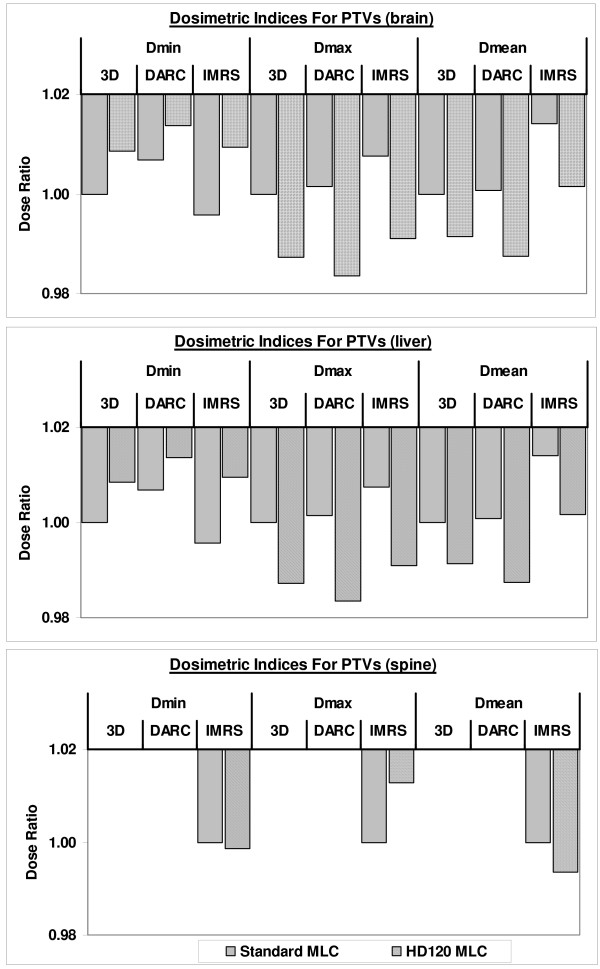
**The percentage difference of PTV coverage between plans using other treatment techniques and MLC systems and the baseline plans, the 3D treatment technique with standard MLC**.

For each case, the PTV coverage was essentially equivalent among different plans. Additional file 2 lists the dosimetric indices for all cases. The small standard deviations for all plans indicate consistency in applying the treatment techniques to achieve the optimal dose coverage; i.e., the prescription isodose covered at least 97% of the target volume and the dose heterogeneity inside the target was kept at about 10%. For each individual case, the D_min _was within 2% and D_max _was within 2.5% among different plans. Using the 3D treatment technique with standard MLC as baseline, Figure 2 displays the percentage difference between plans using other treatment techniques and the HD120 MLC system and the baseline plans.

Compared to the standard MLC system, the HD120 MLC improved the dose homogeneity for the brain group, with larger D_min _(p < 0.01) and smaller D_max _(p < 0.01). In contrast, the HD120 MLC had no significant impact on D_min_, D_max_, or D_mean _values for either the liver group or the spine group. The use of intensity modulation also significantly reduced the D_max _values for the liver group (p <0.01). Although statistically significant, all the above differences were quite small (< 2%) and may likely have little clinical significance.

### OAR dose sparing

**Figure 3:**
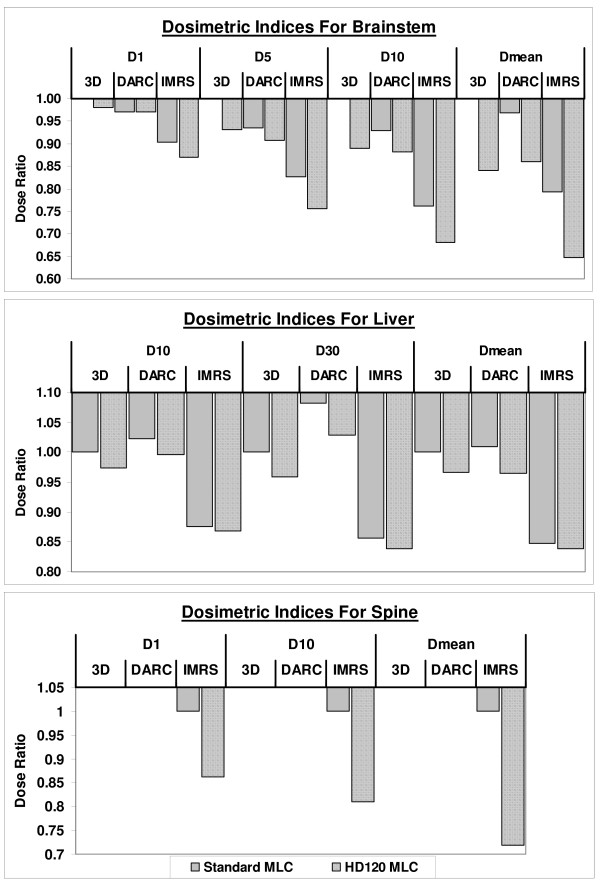
**The percentage difference of OAR sparing between plans using other treatment techniques and MLC systems and the baseline plans, the 3D treatment technique with standard MLC**.

Again, using the 3D treatment technique with the standard MLC as the baseline, Figure 3 displays the percentage difference between the baseline and plans using other treatment techniques and/or the HD120 MLC system

For the brain group, the HD120 MLC and the use of intensity modulation contributed jointly to dose sparing of the brainstem, as a positive interaction of these two factors was detected with GEE (p = 0.003). First, the intensity modulation technique showed significant sparing for the brainstem, compared to other treatment techniques (Additional file 3). Specifically, IMRT produced the greatest dose reduction in the high-dose region, as reflected by the D_1 _and D_5 _doses. The reduction in D_1 _was about 10% and 9%, and in D_5 _was about 18% and 14%, when 3D and DARC techniques, respectively, were replaced by the IMRT technique. Secondly, for the IMRT technique the HD120 MLC also reduced all brainstem doses (p = 0.04) relative to the standard MLC. The improved field shaping with the HD120 MLC also helped to reduce the D_5 _dose for all techniques by 3 – 9% (p = 0.003). Third, the HD120 MLC combined with IMRT jointly benefited dose reduction in the middle dose range, reflected by the D_10 _dose, (p = 0.001). As a result, the mean dose to the brainstem was reduced by 35%, between the best (IMRT with the HD120 MLC) and the worst (3D with standard MLC) plans.

Similarly, the HD120 MLC and the IMRT techniques contributed jointly to the dose sparing for the liver group (Additional file 4). The HD120 MLC reduced doses at both D_10 _and D_30_, as well as the mean dose (p < 0.01). The dose reduction (at all levels) attributable to the HD120 MLC was between 3 – 5% with the 3D and DARC techniques and between 1 – 2% with the IMRT technique. When comparing treatment techniques, IMRT plans were significantly better than either 3D or DARC plans. The IMRT technique improved the dose sparing at all levels, as the D_10_, D_30 _and D_mean _indices were reduced by over 12% over the 3D/DARC techniques.

**Figure 4:**
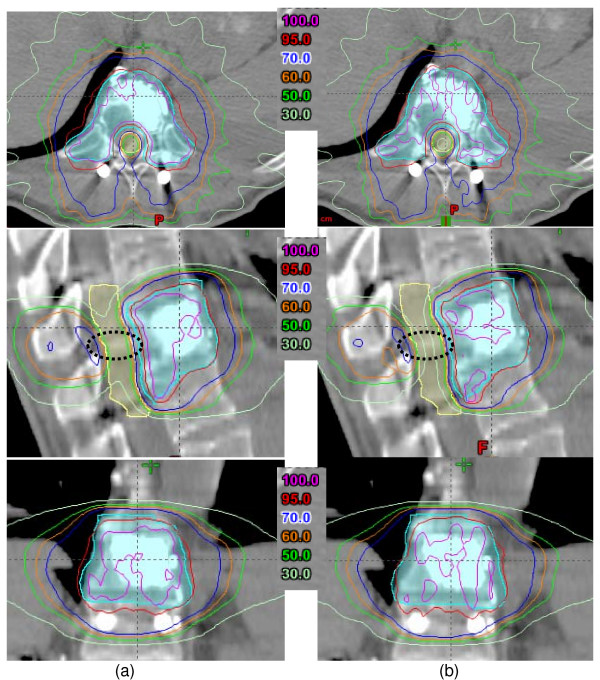
**Example dose distribution of a spine case, IMRT plans with (a) standard MLC system and (b) with HD120 MLC system**. The black circles indicate the regions where dose fall off being significantly different between the two plans.

For the spine group, using the HD120 MLC substantially improved cord sparing (p < 0.01) as the ability to map the dose to the concave-shaped target improved (Additional file 5). Figure 4 displays dose distribution for a spine case. As shown, the dose fall off is much steeper at the target-cord junction for the smaller leaves. The overall dose reduction was 14%, 19% and 29% for the D_1_, D_10 _and D_mean _dose indices, respectively.

### Effect of target shape and volume size

The effect of target volume was compared with the brain and the liver group. As stated earlier, the targets were in general round or ellipsoidal for both the brain and liver lesions, and the most significant differences between these two groups were the target and OAR volumes. The lesions in the brain were several times smaller than those in the liver, and the size of the brainstem was also several times smaller than the liver. The dose reduction (combining all levels) contributed by the HD120 MLC decreased, from 4 – 11% to 1 – 5%, as target sizes increased from the brain group to the liver group.

The effect of target shape was compared with the spine and the liver group. While the target volumes were similar for the liver and the spine groups, the target shapes were much more complex in the spine group (concave vs. round or ellipsoidal for the liver). In contrast to the moderate contribution for liver lesions, the HD120 MLC significantly improved cord sparing for the spine group. By using the 120HD MLC, the dose to the cord was reduced on average by 19% to 14% at high to middle dose levels (D_1 _to D_10_, respectively) for the spine plans.

## Discussion

This study was designed to provide similar target coverage for all plans. The prescription dose was set to the isodose line at the periphery of the PTV that covered at least 97% of the target for all plans. Using this strategy, the DVH to the target volumes was very similar using different treatment planning techniques, yielding dose indices that varied less than 2.5% for the same lesion.

The results from this study suggested that the degree of improved organ sparing varied with target size and shape. The targets in the brain and liver groups are similar in shape (round or ellipsoidal) differed substantially in volume (small vs. large). The lesions in the brain were several times smaller than those in the liver, and the volume of the brainstem was also several times smaller than that of the liver itself. The HD120 MLC yields a better match of the beam aperture to the target projection; however, its benefits become less noticeable as the target/OAR becomes larger. Therefore, the dose reduction contributed by the HD120 MLC decreased, from 4 – 11% to 1 – 5%, as target sizes increased between the brain and liver groups. On the other hand, the targets in the liver and spine groups were of roughly the same size but fell into different shape groups (round vs. concave, respectively). The concave lesion shape presented a challenge for conventional techniques to provide adequate target coverage and optimal organ sparing. Clinically, all spine SBRT lesions in our institution are planned with IMRT, owing to the ability to manipulate the intensity at virtually the voxel level using this technique. Since spinal cords were always adjacent to the target volume, the ability to manipulate the radiation beam with greater precision via the high-definition MLC leaves helped reduce the doses the cord received. Therefore, in contrast to the moderate benefit for liver lesions, the HD120 MLC significantly improved cord sparing for the spine group, realizing a 14%–19% dose reduction in D_1 _and D_10_, respectively.

## Conclusion

The finer HD120 MLC in combination with IMRS provides significant dosimetric benefits for SRS/SBRT. Sparing of the OARs is dependent on the lesion and critical organ size and shape complexity. Small lesions (such as brain lesions treated with SRS) and complex target/OAR geometry (such as the spine lesions encountered in SBRT) will benefit most from the finer-leaf collimator and treatment planning capabilities provided by a dedicated radiosurgery system, compared to larger and more rounded or regularly shaped target volumes. Prospective clinical trials with comprehensive data collection should be conducted to determine whether these dosimetric advantages translate into clinically significant benefits.

## Competing interests

The authors declare that they have no competing interests.

## Authors' contributions

All authors read and approved the final manuscript.

QJW, ZW, FFY and JPK designed the study and the analysis, generated the treatment plans, performed the analysis drafted and revised the manuscript.

ML participated in the study design, statistically analysis and revised the manuscript.

JM and ZC participated in the study design and revised the manuscript.

CH participated in the study design, provided technique assistance and revised the manuscript.

## Supplementary Material

Additional file 1 **Table S1.** Target volumes for the brain, the liver and the spine groups. Click here for file

Additional file 2 **Table S2.** Dosimetrical Indices for PTVs of the brain, liver and spine groups. Click here for file

Additional file 3 **Table S3.** Dosimetrical indices for the five braincases. Click here for file

Additional file 4 **Table S4.** Dosimetrical indices for the five liver cases. Click here for file

Additional file 5 **Table S5.** Dosimetrical indices and statistical comparisons for the five spine cases. Click here for file
