# Supplementary material for: Impact of collimator leaf width and treatment technique on stereotactic radiosurgery and radiotherapy plans for intra- and extracranial lesions
Source: Radiat Oncol. 2009 Jan 21;4:3. doi: 10.1186/1748-717X-4-3 (PMC2637285; doi:10.1186/1748-717X-4-3)
Supplement: Additional file 5 — Table S5. Dosimetrical indices and statistical comparisons for the five spine cases. [file 1748-717X-4-3-S5.doc]

**Table 5. Dosimetrical indices and statistical comparisons for the five spine cases.**

|  | **Cord D_1_**  **(mean + s.d., cGy)** |  |  | **Cord D_10_**  **(mean + s.d., cGy)** |  |  | **Cord D_mean_**  (**mean + s.d., cGy)** |
| --- | --- | --- | --- | --- | --- | --- | --- |
| **standard MLC** | **1120 + 57** |  |  | **984 + 64** |  |  | **723 + 84** |
| **radiosurgery MLC** | **966 + 70** |  |  | **796 + 97** |  |  | **519 + 109** |
