# Supplementary material for: Impact of collimator leaf width and treatment technique on stereotactic radiosurgery and radiotherapy plans for intra- and extracranial lesions
Source: Radiat Oncol. 2009 Jan 21;4:3. doi: 10.1186/1748-717X-4-3 (PMC2637285; doi:10.1186/1748-717X-4-3)
Supplement: Supplementary file 2 [file 1748-717X-4-3-S1.doc]

Table 1. Target volumes for the brain, the liver and the spine groups.

Case #	Site	Target Volume (cc)	Energy
1	Brain #1	2.1	6x
2	Brain #2	2.7	6x
3	Brain #3	3.9	6x
4	Brain #4	7.1	6x
5	Brain #5	12.5	6x
6	Liver #1	17.5	6x
7	Liver #2	29.4	6x
8	Liver #3	39.2	6x,15x
9	Liver #4	62.2	6x,15x
10	Liver #5	251	6x,15x
11	Spine #1	21.81	6x
12	Spine #2	76.95	6x, 15x
13	Spine #3	87.66	6x
14	Spine #4	89.7	6x
15	Spine #5	99.86	6x
