# Supplementary material for: Impact of collimator leaf width and treatment technique on stereotactic radiosurgery and radiotherapy plans for intra- and extracranial lesions
Source: Radiat Oncol. 2009 Jan 21;4:3. doi: 10.1186/1748-717X-4-3 (PMC2637285; doi:10.1186/1748-717X-4-3)
Supplement: Supplementary file 3 [file 1748-717X-4-3-S2.doc]

Table 2. Dosimetrical Indices for PTVs of the brain, liver and spine groups.

		PTV Dmin (cGy) (mean + s.d, cGy)			PTV Dmax (cGy) (mean + s.d, cGy)			PTV Dmean (cGy) (mean + s.d, cGy)		
		3D	DARC	IMRS	3D	DARC	IMRS	3D	DARC	IMRS
Brain	standard MLC	1169 ± 17	1177 ± 7	1164 ± 4	1334 ± 15	1336 ± 8	1344 ± 9	1277 ± 9	1278 ± 6	1295 ± 5
	radiosurgery MLC	1179 ± 9	1185 ± 4	1180 ± 10	1317 ± 14	1312 ± 14	1322 ± 16	1266 ± 8	1261 ± 4	1279 ± 14
Liver	standard MLC	3564 ± 5	3557 ± 18	3552 ± 19	3865 ± 53	3896 ± 59	3788 ± 133	3746 ± 37	3760 ± 20	3740 ± 45
	radiosurgery MLC	3566 ± 4	3547 ± 10	3561 ± 21	3886 ± 44	3891 ± 70	3801 ± 59	3758 ± 27	3753 ± 30	3724 ± 47
Spine	standard MLC			1475 ± 46			1796 ± 24			1690 ± 16
	radiosurgery MLC			1473 ± 48			1819 ± 63			1679 ± 26

3D = 3D static conformal plan, DARC = dynamic conformal arc plan, IMRS = intensity-modulated radiosurgery plan
