# Supplementary material for: Impact of collimator leaf width and treatment technique on stereotactic radiosurgery and radiotherapy plans for intra- and extracranial lesions
Source: Radiat Oncol. 2009 Jan 21;4:3. doi: 10.1186/1748-717X-4-3 (PMC2637285; doi:10.1186/1748-717X-4-3)
Supplement: Supplementary file 4 [file 1748-717X-4-3-S3.doc]

Table 3. Dosimetrical indices for the five braincases.

	Brainstem D_1(cGy) (mean + s.d, cGy)			Brainstem D_5 (cGy) (mean + s.d, cGy)			Brainstem D_{10} (cGy) (mean + s.d, cGy)			Brainstem D_{mean}(cGy) (mean + s.d, cGy)		
	3D	DARC	IMRS	3D	DARC	IMRS	3D	DARC	IMRS	3D	DARC	IMRS
standard MLC	1148 ± 53	1114 ± 42	1038 ± 59	1003 ± 105	938 ± 147	829 ± 149	853 ± 165	793 ± 213	649 ± 202	388 ± 179	376 ± 179	308 ± 162
radiosurgery MLC	1125 ± 47	1114 ± 43	998 ± 69	934 ± 144	911 ± 176	757 ± 161	759 ± 220	752 ± 244	580 ± 204	326 ± 183	334 ± 178	251 ± 147

3D = 3D static conformal plan, DARC = dynamic conformal arc plan, IMRS = intensity-modulated radiosurgery plan
