# Supplementary material for: Impact of collimator leaf width and treatment technique on stereotactic radiosurgery and radiotherapy plans for intra- and extracranial lesions
Source: Radiat Oncol. 2009 Jan 21;4:3. doi: 10.1186/1748-717X-4-3 (PMC2637285; doi:10.1186/1748-717X-4-3)
Supplement: Supplementary file 5 [file 1748-717X-4-3-S4.doc]

Table 4. Dosimetrical indices for the five liver cases.

	Liver D_{10}(cGy) (mean + s.d, cGy)			Liver D_{30} (cGy) (mean + s.d, cGy)			Liver D_{mean}(cGy) (mean + s.d, cGy)		
	3D	DARC	IMRS	3D	DARC	IMRS	3D	DARC	IMRS
standard MLC	2043 ± 660	2091 ± 752	1790 ± 633	1019 ± 423	1102 ± 449	873 ± 440	795 ± 279	803 ± 289	674 ± 261
radiosurgery MLC	1990 ± 685	2035 ± 765	1775 ± 637	977 ± 450	1048 ± 457	855 ± 450	768 ± 297	767 ± 287	667 ± 265

3D = 3D static conformal plan, DARC = dynamic conformal arc plan, IMRS = intensity-modulated radiosurgery plan
